# 固相萃取-高效液相色谱-串联质谱法测定人体血清中26种全氟及多氟烷基化合物

**DOI:** 10.3724/SP.J.1123.2024.10002

**Published:** 2025-09-08

**Authors:** Yingxiao YUE, Yating BIAN, Yufan CHENG, Lu HE, Dan WANG, Peixia YAN, Wei YAN, Guiying LIU, Huan SONG, Liangpo LIU

**Affiliations:** 1.山西医科大学公共卫生学院卫生检验教研室，山西 太原 030001; 1. Department of Public Health Laboratory Sciences，School of Public Health，Shanxi Medical University，Taiyuan 030001，China; 2.山西医科大学煤炭环境致病与防治教育部重点实验室，山西 太原 030001; 2. Key Laboratory of Coal Environmental Pathogenicity and Prevention，Ministry of Education，Shanxi Medical University，Taiyuan 030001，China; 3.山西医科大学黄河流域生态公共卫生安全研究中心，山西 太原 030001; 3. Center for Ecological Public Health Security of Yellow River Basin，Shanxi Medical University，Taiyuan 030001，China; 4.北京市昌平区疾病预防控制中心，北京 102200; 4. Beijing Changping District Center for Disease Prevention and Control，Beijing 102200，China

**Keywords:** HMR-Lipid 96孔固相萃取板, 高效液相色谱-串联质谱法, 全氟及多氟烷基化合物, 人体血清, HMR-lipid 96-well solid-phase extraction plate, high performance liquid chromatography-tandem mass spectrometry （HPLC-MS/MS）, perfluorinated and polyfluoroalkyl compounds （PFASs）, human serum

## Abstract

全氟及多氟烷基化合物（PFASs）是一类由C-F键构成的人造化合物，具有高稳定性和难降解性，还具有生物累积性和多脏器毒性。因此，构建高通量、高灵敏度的检测方法，对于评估人体中PFASs的暴露水平至关重要。本研究采用商品化的高通量HMR-Lipid 96孔固相萃取板，结合高效液相色谱-串联质谱（HPLC-MS/MS），建立了一种简便、高效且能够同时定量检测人体血清中26种PFASs的方法。血清样本采用HMR-Lipid 96孔固相萃取板进行前处理，采用Phenomenex C_18_色谱柱（250 mm×4.6 mm， 5 μm）作为背景污染捕集柱，以Accucore C_18_色谱柱（100 mm×2.4 mm， 2.6 μm）作为分析柱，以2 mmol/L醋酸铵缓冲溶液和甲醇溶液作为流动相进行梯度洗脱。采用电喷雾电离、负离子扫描（ESI^-^）模式，在多反应监测（MRM）模式下进行定量分析。方法学验证结果表明，26种PFASs在0.2~100 ng/mL范围内线性关系良好，相关系数为0.995 1~0.999 9，检出限（LOD）和定量限（LOQ）分别为0.01~0.15 ng/mL和0.02~0.47 ng/mL。在低（5 ng/mL）、中（10 ng/mL）、高（50 ng/mL）3个加标水平下，26种PFASs的回收率为80.1%~119.5%，相对标准偏差（RSD）为0.5%~11.9%。与传统固相萃取方法相比，本研究建立的方法具有灵敏度高、准确性好、操作简便、萃取速度快、试剂消耗少以及所需样品量小等优点，适用于大规模人群生物监测，为准确评估人体中PFASs的暴露情况及其潜在健康风险提供了科学的方法支撑。

## Abstract (en)

Perfluorinated and polyfluoroalkyl compounds （PFASs） represent a category of synthetic chemicals renowned for their environmental persistence. Owing to their hydrophobic， oleophobic， and high-temperature-resistant properties， PFASs are extensively utilized in industrial， agricultural， and civilian sectors， including applications in leather， textiles， flame-retardant materials， lubricants， and coatings， among others. PFASs can accumulate within the human body， exhibiting multi-organ toxicity. Continuous monitoring of PFASs with ambiguous toxicity profiles is vital for evaluating human exposure and associated health risks. Consequently， the establishment of a high-throughput and highly sensitive detection method is of paramount importance for accurately assessing the exposure levels of PFASs in the human body. In this study， a commercial high-throughput HMR-Lipid 96-well solid-phase extraction plate was adopted， combined with high performance liquid chromatography-tandem mass spectrometry （HPLC-MS/MS）， to establish a simple， efficient method that can simultaneously quantitatively detect 26 PFASs in human serum. Serum samples were extracted using the HMR-lipid 96-well solid-phase extraction plate. The Phenomenex C_18_ chromatography column （250 mm×4.6 mm， 5 μm） was used as the capture column and connected between the liquid chromatography mixer and the autosampler to avoid high background pollution. The target compounds were separated by the Accucore C_18_ chromatography column （100 mm×2.4 mm， 2.6 μm） and analyzed using the electrospray ionization with negative ion scanning mode and multiple reaction monitoring （MRM） mode. The methodological validation results indicated that the 26 PFASs had good linear relationships within the range of 0.2-100 ng/mL， with correlation coefficients （*r*） of 0.995 1-0.999 9. The limits of detection （LODs） and quantification （LOQs） were 0.01-0.15 ng/mL and 0.02-0.47 ng/mL， respectively. At three spiked levels of low， medium and high， the recoveries of the 26 PFASs ranged from 80.1% to 119.5%， and the relative standard deviations （RSDs） ranged from 0.5% to 11.9%. This method has the advantages of high sensitivity， good accuracy， simple operation， fast extraction speed， low reagent consumption and small sample volume required. It is suitable for large-scale population biological monitoring and provides a scientific method support for accurately assessing the exposure of PFASs in the human body and its potential health risks.

全氟及多氟烷基化合物（PFASs）是一类具备热稳定性和化学稳定性的人造化合物，在自然环境中极难发生降解，因而被称作永久化学品^［1］^。PFASs自出现至今已近70年，目前有超过1 400种PFASs被广泛应用于各个领域，涵盖食品包装材料、消防泡沫、快餐容器、防污织物、不沾涂层以及灭火泡沫等^［2-4］^。这些化合物主要通过工业排放^［5］^、大气传输^［6］^、水体扩散^［7］^、土壤渗透^［8］^以及室内粉尘沉降^［9］^等途径进入人体。鉴于传统PFASs具有多脏器毒性^［10］^，我国已对部分PFASs实施限制措施^［11］^。为满足工业需求，开发并应用其替代品是应对全球PFASs监管要求的必要举措。目前，全氟辛酸（PFOA）和全氟辛烷磺酸钾（PFOS）已在人体血液^［12］^、尿液^［13］^、头发^［14］^、胎盘组织^［15］^、精液^［16］^、母乳^［17］^等多种生物介质中被检出。新型PFASs不断涌现，但其人体暴露水平仍不明确。流行病学研究显示，PFASs及其新型替代物具有生殖毒性、内分泌毒性以及神经毒性^［18-20］^，且与乳腺癌患病风险增加有关^［21］^。为及时识别和预防PFASs暴露引发的健康风险并评估其暴露水平，建立简便、高效的PFASs检测方法至关重要。

目前，针对人体血清中PFASs的检测，主要采用液液萃取（LLE）^［22］^和固相萃取（SPE）^［23］^方法进行前处理，高效去除磷脂，并结合液相色谱-串联质谱法（LC-MS/MS）进行定量分析^［24-26］^。LLE作为一种经典的前处理技术，具有操作简便、无需特殊装置的优点，通过选择合适的萃取剂，可实现对特定化合物的选择性提取。例如，Liu等^［25］^以甲基叔丁基醚（MTBE）为萃取液，提取人体血清中的PFASs，并建立了LC-MS/MS检测方法。然而，该方法需对样品进行蛋白沉淀和碱化处理，并反复加萃取液萃取3次，过程繁琐耗时，且易引发交叉污染。随着现代样品前处理技术的发展，SPE技术已广泛应用于环境分析、生物样品分析、食品分析等领域，成为一种高效的样品前处理手段，能够快速实现样品的富集和净化。目前，亲水亲脂平衡固相萃取（SPE-HLB）和弱阴离子交换固相萃取（SPE-WAX）技术是常用的SPE方法^［27］^，但通常需要对萃取柱进行活化、平衡和淋洗除杂等步骤，导致样品消耗量大且有机试剂用量较多。HMR-Lipid 96孔固相萃取板作为一种创新型的无机固相萃取材料，采用通过式净化处理方式，无需进行活化、平衡、淋洗等步骤，操作过程简便、快捷。同时，通过在高比表面积的无机骨架上修饰对磷脂具有选择性吸附作用的官能团，HMR-Lipid 96孔固相萃取板能够去除基质干扰的同时不会吸附目标分析物，净化效果好。因此，HMR-Lipid 96孔固相萃取板在人体生物样品中PFASs及其他污染物的前处理方面展现出潜在的应用前景。

基于此，本研究采用商品化的高通量HMR-Lipid 96孔固相萃取板，结合高效液相色谱-串联质谱（HPLC-MS/MS），建立了一种简便、高效且能够同时测定人体血清中26种PFASs的方法，以期为准确评估人体中PFASs的暴露情况及其潜在健康风险提供科学的方法支撑。

## 1 实验部分

### 1.1 仪器、试剂与材料

高效液相色谱-串联质谱仪（LC-MS/MS 8050，日本Shimadzu公司）；固相萃取正压装置（美国Waters公司）；96孔氮吹仪、Anavo HMR-Lipid超效分体96孔固相萃取板（10 mg/1 mL，北京纳鸥科技有限公司）；台式高速离心机（德国Hettich公司）；涡旋振荡仪（北京大龙兴创实验仪器股份公司）；XX40真空过滤系统、0.45 μm水系滤膜（德国Merck Millipore公司）；GM-0.33A隔膜真空泵（天津市津腾实验设备有限公司）。

26种PFASs标准品（纯度88.7%~100%）：PFOA、PFOS、全氟丁酸（PFBA）、全氟己酸（PFHxA）、全氟壬酸（PFNA）、全氟癸酸（PFDA）、全氟十一烷酸（PFUdA）、全氟十二烷酸（PFDoA）、全氟己烷磺酸钾（PFHxS）、全氟庚烷磺酸钠（PFHpS）、全氟十三烷酸（PFTriDA）、全氟十四烷酸（PFTeDA）、全氟戊酸（PFPeA）、全氟庚酸（PFHpA）、全氟丁基磺酸钾（PFBS）、全氟戊烷磺酸钠（PFPeS）、辛基磺酰胺（PFOSA）、全氟壬烷磺酸钠（PFNS）、全氟癸烷磺酸钠（PFDS）、己烷磺酸钠（4∶2 FTS）、辛烷磺酸钠（6∶2 FTS）、癸烷磺酸钠（8∶2 FTS）、2-（*N*-乙基全氟辛烷磺酰氨基）乙酸（*N*-EtFOSAA）和2-（*N*-甲基全氟辛烷磺酰氨基）乙酸（*N*-MeFOSAA）购于美国Accustandard公司；6∶2氯代多氟醚基磺酸（6∶2 Cl-PFESA）和8∶2氯代多氟醚基磺酸（8∶2 Cl-PFESA）购于北京百灵威科技有限公司。9种PFASs同位素内标（纯度均为98%以上）：^13^C_4_-PFBA、^13^C_2_-PFHxA、^13^C_4_-PFOA、^13^C_5_-PFNA、^13^C_2_-PFDA、^13^C_2_-PFUdA、^13^C_2_-PFDoA、^18^O_2_-PFHxS和^13^C_4_-PFOS购于北京百灵威科技有限公司。乙腈和甲醇（均为HPLC级，美国Honeywell公司）；醋酸铵（分析纯，美国Alfa Aesar公司）；屈臣氏水（广州屈臣氏食品饮料有限公司）。

本研究已经获得山西医科大学医学伦理委员会批准（批准文件编号：2023SJL35）。人群招募及血清样品采集：研究招募了某地区的25名成人志愿者，并向所有志愿者告知了研究目的，整个研究过程严格遵守伦理准则，25名志愿者均签署了知情同意书。采用不含抗凝剂的采血管采集血液，采集后于4 ℃下静置4 h，在4 000 r/min下离心10 min，取出上清液，于-80 ℃条件下保存。

### 1.2 溶液的配制

混合标准溶液：准确称取各PFASs标准品，用甲醇溶解并定容，配制成质量浓度为500 ng/mL的26种PFASs混合标准储备溶液；之后用甲醇逐级稀释混合标准储备溶液，配制成系列质量浓度（10、20、100 ng/mL）的26种PFASs混合标准溶液。

混合内标储备溶液：准确称取各内标标准品，用甲醇溶解并定容，配制成质量浓度为50 ng/mL的9种PFASs混合内标储备溶液。

混合标准工作液：分别准确移取100 μL混合标准储备溶液和100 μL混合内标储备溶液，置于1.5 mL离心管中，加入300 μL的50%甲醇水溶液，得到含有26种PFASs和9种内标的混合标准中间液，其中PFASs的质量浓度均为100 ng/mL，内标的质量浓度均为10 ng/mL；用50%甲醇水溶液逐级稀释混合标准中间液，配制成质量浓度分别为0、0.2、1、5、10、25、50、100 ng/mL的系列混合标准工作液，其中内标的质量浓度均为10 ng/mL。将配制好的混合标准工作液转移至聚丙烯进样瓶中，于4 ℃下保存。

2 mmol/L 醋酸铵缓冲溶液（pH 7.0）：准确称取154.0 mg醋酸铵，置于1 L容量瓶中，用蒸馏水溶解并定容，混匀后用0.45 μm滤膜过滤，并于4 ℃下保存。

### 1.3 样品前处理

采用HMR-Lipid 96孔固相萃取板对血清样品中的目标分析物进行提取，具体步骤如下：首先，将血清样品从-80 ℃冰箱中取出，置于4 ℃环境下解冻。解冻后，移取50 µL血清至1.5 mL离心管中，随后加入50 µL同位素内标混合标准溶液（10 ng/mL），充分混匀后，将离心管置于4 ℃条件下静置1 h；接着，在5 500 r/min下离心3 min，将上清液全部转移至HMR净化管中，然后沿净化管壁加入200 µL乙腈，静置5 min；采用正压装置对净化管施加700 Pa的压力，待管内液体逐滴滴下，再次沿管壁加入200 µL乙腈，并继续施加正压，直至净化管内无液体残留；将收集到的洗脱液在40 ℃下氮吹至干，加入50 µL的50%甲醇水溶液复溶，涡旋混匀后转移至进样瓶（带塑料内衬管）中，待测定。

### 1.4 仪器分析条件

#### 1.4.1 色谱条件

将Phenomenex C_18_色谱柱（250 mm×4.6 mm，5 μm，美国Phenomenex公司）连接在液相色谱混合器与自动进样器之间，用作捕集柱；选用Accucore C_18_色谱柱（100 mm×2.4 mm，2.6 μm，美国Thermo Scientific公司）作为分析柱，并在分析柱前端连接Accucore C_18_保护柱（10 mm×4 mm，5 μm，美国Thermo Scientific公司）；柱温箱温度为35 ℃；进样体积为5.0 μL；洗脱速率为0.45 mL/min。流动相A为2 mmol/L醋酸铵缓冲溶液，流动相B为甲醇，采用线性梯度洗脱方式，具体洗脱程序见表1。

**表1:** 梯度洗脱程序

Time/min	Elution velocity/（mL/min）	*φ*（A）/%	*φ*（B）/%
0	0.45	70	30
5.0	0.45	0	100
15.0	0.45	0	100
15.5	0.45	70	30
25.0	0.45	70	30

A： 2 mmol/L ammonium acetate buffer solution； B： methanol.

#### 1.4.2 质谱条件

离子源：电喷雾电离（ESI）源；扫描方式：负离子扫描；接口电压：3 kV；脱溶剂温度：250 ℃；接口温度：300 ℃；加热块温度：400 ℃；加热气体流量：10.0 L/min；雾化气体流量：3.0 L/min；干燥气体流量：10.0 L/min；驻留时间：20 ms。采用多反应监测（MRM）模式进行定量分析。质谱参数详见表2。

**表 2:** 26种PFASs及9种同位素内标的保留时间和质谱参数

Compound	*M*_r_/（g/mol）	*t*_R_/min	Transition	CE/eV	IS
Perfluoro-*n*-butanoic acid （PFBA）	214.04	4.65	213.05>169.00^*^	12	^13^C_4_-PFBA
Perfluoro-*n*-pentanoic acid （PFPeA）	264.65	10.00	263.05>218.95^*^	9	^13^C_2_-PFHxA
Potassium perfluoro-1-butanesulfonate （PFBS）	300.00	10.26	299.00>80.00^*^	35	^18^O_2_-PFHxS
Perfluoro-*n*-hexanoic acid （PFHxA）	314.05	10.75	313.05>269.05^*^	10	^13^C_2_-PFHxA
Sodium perfluoro-1-pentanesulfonate （PFPeS）	350.00	10.81	349.00>80.00^*^	45	^18^O_2_-PFHxS
			349.00>99.00	31	
Sodium 1*H*，1*H*，2*H*，2*H* -perfluoro-1-hexanesulfonate （4∶2 FTS）	328.00	10.88	327.00>307.00^*^	19	^13^C_4_-PFOS
Sodium perfluoro-1-heptanesulfonate （PFHpS）	450.00	11.00	449.00>80.00^*^	49	^18^O_2_-PFHxS
			449.00>99.00	39	
Perfluoro-*n*-heptanoic acid （PFHpA）	364.06	11.13	363.05>319.10^*^	11	^13^C_4_-PFOA
			363.05>119.20	22	
Potassium perfluoro-1-hexanesulfonate （PFHxS）	400.00	11.13	399.00>79.95^*^	46	^18^O_2_-PFHxS
			399.00>99.00	35	
Sodium 1*H*，1*H*，2*H*，2*H*-perfluoro-1-octanesulfonate （6∶2 FTS）	450.15	11.40	449.15>80.00^*^	45	^13^C_4_-PFOS
			449.15>99.00	38	
Perfluoro-*n*-octanoic acid （PFOA）	414.07	11.40	413.05>369.00^*^	11	^13^C_4_-PFOA
			413.05>169.05	19	
Perfluoro-*n*-nonanoic acid （PFNA）	464.08	11.63	463.05>419.00^*^	12	^13^C_5_-PFNA
			463.05>218.90	19	
Potassium perfluoro-1-octanesulfonate （PFOS）	500.00	11.63	499.00>80.00^*^	49	^13^C_4_-PFOS
			499.00>99.05	41	
6∶2 chlorinated polyfluorinated ether sulfonate acid （6∶2 Cl-PFESA）	531.58	11.73	530.55>350.90^*^	27	^13^C_4_-PFOS
			530.55>83.05	30	
Sodium perfluoro-1-nonanesulfonate （PFNS）	550.00	11.82	549.00>79.90^*^	55	^13^C_4_-PFOS
			549.00>98.85	46	
Perfluoro-*n*-decanoic acid （PFDA）	514.08	11.83	513.05>468.95^*^	13	^13^C_2_-PFDA
			513.05>218.95	18	
Sodium perfluoro-1-decanesulfonate （PFDS）	600.00	12.00	599.00>79.90^*^	55	^13^C_4_-PFOS
			599.00>99.10	52	
Sodium 1*H*，1*H*，2*H*，2*H*-perfluoro-1-decanesulfonate （8∶2 FTS）	528.00	12.01	527.00>506.95^*^	27	^13^C_4_-PFOS
Perfluoro-*n*-undecanoic acid （PFUdA）	564.09	12.01	563.10>519.00^*^	13	^13^C_2_-PFUdA
			563.10>269.10	20	
8∶2 chlorinated polyfluorinated ether sulfonate acid （8∶2 Cl-PFESA）	631.60	12.07	630.60>450.90^*^	30	^13^C_4_-PFOS
			630.60>83.05	45	
*N*-Methylperfluorooctanesulfonamidoacetic acid （*N*-MeFOSAA）	571.00	12.12	570.00>418.85^*^	22	^13^C_4_-PFOA
			570.00>219.00	27	
Perfluoro-*n*-dodecanoic acid （PFDoA）	614.10	12.17	613.10>568.95^*^	14	^13^C_2_-PFDoA
			613.10>169.20	29	
*N*-Ethylperfluorooctanesulfonamidoacetic acid （*N*-EtFOSAA）	585.24	12.22	584.25>419.00^*^	22	^13^C_2_-PFUdA
			584.25>218.90	20	
Perfluoro-*n*-tridecanoic acid （PFTriDA）	664.10	12.31	663.10>618.90^*^	13	^13^C_2_-PFDoA
			663.10>269.20	20	
Perfluoro-*n*-tetradecanoic acid （PFTeDA）	714.11	12.44	713.10>668.85^*^	15	^13^C_2_-PFDoA
			713.10>218.90	25	
Perfluorooctane sulfonamide （PFOSA）	499.15	13.05	498.15>78.00^*^	45	^13^C_4_-PFOS
			498.15>169.05	30	
^13^C_4_-PFBA	218.00	4.64	217.00>172.05^*^	12	/
^13^C_2_-PFHxA	316.00	10.75	315.00>270.00^*^	11	/
^18^O_2_-PFHxS	404.00	11.14	403.00>84.00^*^	43	/
^13^C_4_-PFOA	418.00	11.40	417.00>371.90^*^	12	/
^13^C_4_-PFOS	504.00	11.63	503.00>80.00^*^	53	/
^13^C_5_-PFNA	469.00	11.64	468.00>423.00^*^	12	/
^13^C_2_-PFDA	516.00	11.83	515.00>469.95^*^	13	/
^13^C_2_-PFUdA	566.00	12.02	565.00>519.85^*^	15	/
^13^C_2_-PFDoA	616.00	12.17	615.00>570.00^*^	15	/

* Quantitative ion pair； CE： collision energy； /： no value.

## 2 结果与讨论

### 2.1 仪器条件优化

#### 2.1.1 质谱参数优化

采用自动进样器分别对26种PFASs混合标准储备溶液（500 ng/mL）及9种PFASs混合内标储备溶液（50 ng/mL）进样分析，以优化质谱条件。实验选取全扫描（Q1 Scan）模式，设置流速为0.2 mL/min，柱温箱温度为35 ℃，检测时间为2 min。在此条件下，分别对母离子、子离子、碰撞能量等参数进行自动优化，挑选信号响应较高且干扰较小的碎片离子作为定量子离子，最终确定了26种PFASs及9种同位素内标的质谱条件（表2）。

#### 2.1.2　色谱条件优化

预实验结果发现，PFOS、PFHxS和PFHpA存在较高的背景值，这会对待测物的含量分析产生干扰。已有研究报道了全氟类化合物LC-MS/MS检测过程中仪器污染的处理方法，即在混合器和自动进样器之间连接一根捕集柱，以此达到排除背景污染的目的，该方法所获得的加标回收率为66.8%~111.9%^［28］^。本研究旨在排除仪器背景干扰的同时进一步提高方法的灵敏度与准确度，从而实现人体血清中PFASs更准确的定量分析。为此，实验分别选用Diamonsil C_18_（2）色谱柱（250 mm×4.6 mm，5 μm，北京迪科马科技有限公司）、Agilent C_18_色谱柱（250 mm×4.6 mm，5 μm，美国Agilent Technologies公司）和Phenomenex C_18_色谱柱（250 mm×4.6 mm，5 μm）作为捕集柱开展预实验，考察它们对背景污染的去除效果以及对待测物的分离效果。经实验发现，与另两种色谱柱相比，当采用Phenomenex C_18_色谱柱作为背景污染捕集柱时，能够有效消除背景污染（流动相为100%有机相），实现对目标待测物的准确测定。

在1.4节条件下，26种PFASs的总离子色谱图见图1，可以看出，26种PFASs的色谱峰形和响应强度均较为理想。

**图1:**
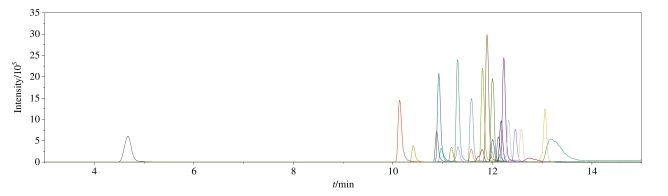
26种PFASs混合标准溶液（25 ng/mL）的总离子色谱图

### 2.2 方法学考察

#### 2.2.1 线性范围、回归方程、检出限与定量限

采用本方法对系列质量浓度的混合标准工作液进行LC-MS/MS分析。以目标待测物与对应同位素内标的定量离子峰面积之比为纵坐标（*Y*），目标待测物与对应同位素内标的质量浓度之比为横坐标（*X*），绘制相应的标准曲线。结果显示，26种PFASs在0.2~100 ng/mL范围内线性关系良好，相关系数（*r*）为0.995 1～0.999 9。在低质量浓度校准曲线下确定检出限（LOD）和定量限（LOQ），用3倍信噪比（*S/N*）计算LOD，10倍*S/N*计算LOQ。结果表明，26种PFASs的LOD和LOQ分别为0.01~0.15 ng/mL和0.02~0.47 ng/mL，相关数据见表3。与Liu等^［25］^报道的血清中24种PFASs的检测方法（LOD和LOQ分别为0.01~0.25 ng/mL和0.03~0.75 ng/mL）相比，本方法的LOD和LOQ更低，说明本方法更加灵敏。

**表3:** 26种PFASs的回归方程、相关系数、线性范围、检出限和定量限

Compound	Regression equation	*r*	Linear range/（ng/mL）	LOD/（ng/mL）	LOQ/（ng/mL）
PFBA	*Y*=0.91*X*+0.0033	0.9999	0.2-100	0.13	0.38
PFHxA	*Y*=0.84*X*+0.0035	0.9996	0.2-100	0.03	0.10
PFOA	*Y*=0.73*X*+0.0033	0.9989	0.2-100	0.08	0.23
PFNA	*Y*=0.88*X*+0.0057	0.9958	0.2-100	0.03	0.09
PFDA	*Y*=0.84*X*+0.0028	0.9971	0.2-100	0.03	0.08
PFUdA	*Y*=0.53*X*-0.0008	0.9981	0.2-100	0.04	0.12
PFDoA	*Y*=0.53*X*-0.0001	0.9988	0.2-100	0.04	0.12
PFHxS	*Y*=0.95*X*+0.0094	0.9998	0.2-100	0.05	0.15
PFHpS	*Y*=0.81*X*-0.0025	0.9990	0.2-100	0.11	0.35
PFOS	*Y*=0.98*X*+0.0045	0.9951	0.2-100	0.13	0.41
PFTriDA	*Y*=0.37*X*+0.0026	0.9964	0.2-100	0.04	0.11
PFTeDA	*Y*=0.37*X*-0.0047	0.9956	0.2-100	0.04	0.13
PFPeA	*Y*=0.70*X*+0.0062	0.9981	0.2-100	0.05	0.15
PFHpA	*Y*=1.04*X*+0.0010	0.9994	0.2-100	0.09	0.27
PFBS	*Y*=0.96*X*+0.0001	0.9997	0.2-100	0.05	0.15
6∶2 Cl-PFESA	*Y*=6.45*X*+0.0038	0.9999	0.2-100	0.01	0.03
8∶2 Cl-PFESA	*Y*=5.11*X*-0.0072	0.9996	0.2-100	0.01	0.03
PFPeS	*Y*=0.80*X*+0.0007	0.9998	0.2-100	0.15	0.47
PFOSA	*Y*=2.80*X*+0.0096	0.9983	0.2-100	0.01	0.03
PFNS	*Y*=0.50*X*+0.0016	0.9984	0.2-100	0.10	0.29
PFDS	*Y*=0.41*X*-0.0022	0.9997	0.2-100	0.07	0.22
4∶2 FTS	*Y*=1.57*X*+0.0059	0.9978	0.2-100	0.02	0.06
6∶2 FTS	*Y*=0.67*X*-0.0007	0.9986	0.2-100	0.15	0.44
8∶2 FTS	*Y*=1.16*X*+0.0048	0.9962	0.2-100	0.02	0.07
*N*-EtFOSAA	*Y*=0.16*X*+0.0007	0.9983	0.2-100	0.01	0.02
*N*-MeFOSAA	*Y*=0.29*X*-0.0010	0.9993	0.2-100	0.01	0.05

*Y*： peak area ratio of PFASs to ISs； *X*： mass concentration ratio of PFASs to ISs.

#### 2.2.2 回收率和精密度

通过在空白血清中添加不同水平（5、10、50 ng/mL）的26种PFASs混合标准工作液来考察方法的准确度与精密度。每个加标水平设置6个平行样本，同时设置质量控制样品（程序空白组和血清空白组）。将质量控制样品与加标血清样品一同进行处理，并在加标血清样本测定过程中穿插进行质量控制样品的LC-MS/MS分析，以此来评估方法的准确度与精密度。结果表明，在3个加标水平下，26种PFASs的加标回收率为80.1%~119.5%，相对标准偏差（RSD）为0.5%~11.9%，具体结果见表4。与Liu等^［25］^报道的血清中24种PFASs检测方法相比（加标回收率为64.0%~124%，RSD为0.74%~11.2%），本方法具有更高的准确度。

**表4:** 26种PFASs的回收率和相对标准偏差（*n*=6）

Compound	5 ng/mL		10 ng/mL		50 ng/mL		Compound	5 ng/mL		10 ng/mL		50 ng/mL	
	Recovery/%	RSD/%	Recovery/%	RSD/%	Recovery/%	RSD/%		Recovery/%	RSD/%	Recovery/%	RSD/%	Recovery/%	RSD/%
PFBA	93.2	4.8	91.1	3.5	93.9	1.6	PFHpA	87.3	1.6	86	3.6	82.4	0.7
PFHxA	98.6	4.6	96.5	5	91.6	2	PFBS	99.7	4.2	98.6	1.1	99.4	2.7
PFOA	92.4	0.6	85.3	4.1	85.8	2.7	6∶2 Cl-PFESA	93.3	4	87.3	4.6	86.1	1
PFNA	90.5	4.3	89.4	2.6	87	1.3	8∶2 Cl-PFESA	111.2	3.1	100.2	11.9	101.2	3.9
PFDA	81	3	80.1	3.5	82	3.3	PFPeS	98.2	1.3	93	3.2	89.7	3.3
PFUdA	92.1	8.5	100.4	11.6	91.6	1.2	PFOSA	80.9	11	80.9	9	83.7	7.3
PFDoA	100.6	11.9	104.6	8.7	98.1	2.9	PFNS	96.5	10.9	85.4	9.5	91.9	0.9
PFHxS	101	5.3	100.8	4.1	94.7	1.8	PFDS	107.5	7.5	92.2	7.9	99.7	4.8
PFHpS	103.4	2.8	96.8	3.9	94.9	1.9	4∶2 FTS	114.4	6.2	111.5	2.8	106.7	5.2
PFOS	91.1	3.6	93.6	0.5	86.9	2.3	6∶2 FTS	93.1	5.5	96.9	4.2	87.1	2
PFTriDA	114.8	2.6	118.3	7	111.6	3.2	8∶2 FTS	102.1	1	93	7.6	84.1	1.3
PFTeDA	112	8.7	116.1	2.3	115.9	5.5	*N*-EtFOSAA	116.4	5	119.5	9.7	119.2	4.3
PFPeA	102.3	7.8	96.9	2.4	98	2	*N*-MeFOSAA	108.9	3.6	103.1	9.6	106.5	4.4

### 2.3 不同检测方法比较

将本方法与已报道的PFASs前处理方法进行比较，比较参数包括血清样本用量、萃取时间、有机试剂用量、LOD、LOQ和回收率等，具体见表5。Liu等^［25］^采用经典的LLE前处理方式，在前处理过程中，血清样本需分别进行蛋白沉淀和碱化处理，之后用MTBE反复萃取3次，有机试剂用量大且前处理耗时较长；Gao等^［29］^采用乙腈沉淀法从血清样品中提取PFASs，该方法虽能够沉淀蛋白质，但无法完全去除样品中的其他干扰物（如脂类和色素等），后续还需对样品进行进一步纯化，所得到的LOQ较高；Yan等^［30］^和杨觅等^［23］^分别采用Oasis HLB固相萃取和Oasis WAX固相萃取方法对血清中的PFFASs进行前处理，这两种方法均需对萃取柱进行活化、平衡和淋洗除杂等步骤，前处理耗时较长且有机试剂用量较大。相比之下，本研究所建立的HMR-Lipid 96孔固相萃取法仅需50 μL血清样品，且无需活化和平衡操作。HMR-Lipid 96孔固相萃取板具备通过式净化的优势，能一步完成蛋白沉淀和磷脂去除，具有净化高效、操作简便、前处理耗时短、准确度与精密度良好等特点，是一种高通量且易于操作的血清中PFASs前处理方法。

**表5:** 血清样品中 PFASs的前处理方法比较

Pretreatment method	Numbers of PFASs	Sample dosage/μL	Pretreatment time/h	Organic reagent dosage/mL	LOD/ （ng/mL）	LOQ/ （ng/mL）	Recovery/%	Ref.
LLE	24	500	2.5	1400	0.01-0.25	0.03-0.75	64.0-124.0	［^25^］
Acetonitrile precipitation	46	50	0.4	10	-	0.10-1	68.9-115.7	［^29^］
Oasis HLB SPE	33	100	1.5	332	0.002-0.016	0.006-0.051	80.1-116.0	［^30^］
Oasis WAX SPE	12	500	1.5	1575	0.01-0.02	0.03-0.07	76.0-110.2	［^23^］
HMR-Lipid SPE	26	50	0.5	39	0.01-0.15	0.02-0.47	80.1-119.5	this study

-： not given.

### 2.4 实际样品检测

为进一步评估该方法在实际样品测定中的可行性，将该方法应用于25份人体血清样本的测定。结果如表6所示，26种PFASs中有10种PFASs的检出率为100%，包括PFOA、PFOS、PFHxS、PFNA、PFDA、PFBA、6∶2 Cl-PFESA、PFHpS、PFPeA和PFUdA；PFTriDA的检出率为60%，6∶2 FTS检出率为52%，PFPeS的检出率为24%，其余13种PFASs未检出。尽管PFOS和PFOA分别于2009年和2019年相继在全球范围内被禁用，但由于它们具有生物蓄积性和较长的半衰期^［31］^，PFOS（检出平均水平为5.46 ng/mL）和PFOA（检出平均水平为7.67 ng/mL）仍是人体血清中检出率和检出水平最高的PFASs。Wen等^［32］^对美国成年人血清中PFASs的检测结果也显示，PFOS和PFOA是暴露水平最高的PFASs。此外，6∶2 Cl-PFESA是除PFOS和PFOA以外人体中暴露水平最高的PFASs污染物，其平均暴露水平达到1.25 ng/mL。目前，PFASs对人类健康的风险已成为学术研究热点，然而对于新型PFASs的环境存在、人体暴露及健康风险，我们仍缺乏足够的认识。本研究所建方法可为人体污染物暴露与健康风险评估提供可靠的检测技术支持。

**表6:** 人体血清样本中PFASs的测定结果（*n*=25） (ng/mL)

Compound	Arithmetic mean （SD）	Geometric mean	Minimum	25th	50th	75th	Maximum	Detection rate/%
PFOA	7.67 （1.31）	7.56	5.96	6.35	7.98	8.83	9.61	100
PFOS	5.46 （2.59）	4.80	2.54	2.69	6.86	7.78	8.76	100
PFHxS	0.95 （0.40）	0.87	0.49	0.56	0.94	1.40	1.60	100
PFNA	0.88 （0.27）	0.84	0.55	0.61	0.87	1.14	1.29	100
PFDA	0.66 （0.15）	0.64	0.43	0.51	0.68	0.77	0.91	100
PFBA	0.08（0.01）	0.08	0.07	0.08	0.08	0.09	0.10	100
PFUdA	0.42 （0.07）	0.41	0.33	0.35	0.39	0.47	0.59	100
6∶2Cl-PFESA	1.25 （0.53）	1.14	0.66	0.78	1.24	1.73	2.37	100
PFHpS	0.24 （0.12）	0.22	0.11	0.15	0.23	0.30	0.57	100
PFPeA	0.90 （0.12）	0.89	0.64	0.81	0.94	0.99	1.11	100
PFTriDA	0.16 （0.05）	0.15	0.10	0.12	0.15	0.17	0.28	60
6∶2FTS	0.28 （0.09）	0.26	0.15	0.18	0.32	0.34	0.40	52
PFPeS	0.22 （0.04）	0.22	0.17	0.18	0.22	0.26	0.27	24

SD： standard deviation.

## 3 结论

本研究将高通量的HMR-Lipid 96孔固相萃取板与LC-MS/MS技术结合，建立了一种可同时测定人体血清中26种PFASs的方法。该方法具有操作简便、快速的特点，同时具备高灵敏度、良好的准确度与精密度，可应用于大批量实际血清样品的检测，能为全面评估人体内新型PFASs的暴露情况与健康风险提供方法学支持。
